# Congenital retinal macrovessel associated with central retinal vein occlusion in the contralateral eye: a case report

**DOI:** 10.5935/0004-2749.2022-0219

**Published:** 2025-02-11

**Authors:** Matheus Bernardo Costa Leal Arnaut, Mariama de Almeida Ferreira, Fernando Henrique Flores Teixeira, Vanessa Dias Rocha, Ana Clara Thomaz Almeida de Oliveira Rangel, Vitor Barbosa Cerqueira

**Affiliations:** 1 Cirurgia Ocular de São Cristovão, Rio de Janeiro, RJ, Brazil

Dear Editor,

In 1869, Mauthner described a large, aberrant retinal vessel across the macular region^(1)^. In 1982, Brown et al.^(2)^ coined the term “congenital retinal macrovessel” (CRM) in a report of seven cases of an abnormal retinal vessel, typically a vein, crossing the central macula with a vascular distribution above and below the horizontal raphe.

CRM, usually detected on routine examination, is a rare condition, with an approximate prevalence of one in 200,000 people^(3)^. It is markedly unilateral and can be an isolated venous (more commonly) or arterial lesion, or even associated with an artery and a vein, including a cilioretinal artery^(4)^. It usually does not affect vision and is stable on longitudinal follow-up.

This study reports a case of CRM with preserved vision and central retinal vein occlusion in the contralateral eye.

**Figure 1:**
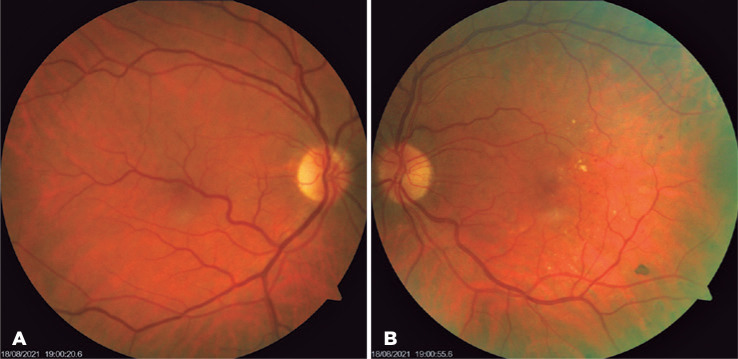
(A) Color retinography of the right eye showing an inferotemporal macrovessel crossing the fovea. (B) Color retinography of the left eye showing hard exudates and microbleeds in the posterior pole.

A 68-year-old man with hypertension and type II diabetes visited the ophthalmic outpatient clinic with a complaint of reduced visual acuity in the left eye. His corrected visual acuity was 20/20 and 20/40, and his intraocular pressure was 13 and 15 mmHg in the right eye (RE) and left eye (LE), respectively. The anterior segment showed no changes on examination. The initial funduscopic examination showed an inferotemporal macrovessel crossing the fovea in the RE and hard exudates, microbleeds, and changed foveal reflex in the LE (Figure 1).

**Figure 2:**
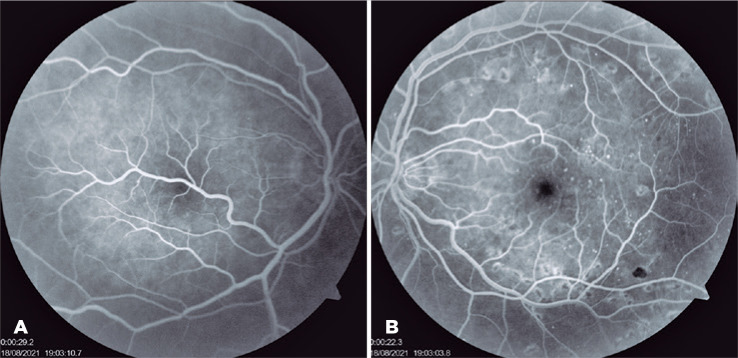
(A) Fluorescein angiography of the right eye showing macrovessel tributaries with normal angiographic flow and a preserved capillary network. (B) Fluorescein angiography of the left eye after treatment showing microaneurysms and panretinal photocoagulation marks.

He was diagnosed with central retinal vein occlusion associated with macular edema in the LE and was treated with argon laser panretinal photocoagulation and 3-monthly injections of anti-vascular endothelial growth factor (anti-VEGF). Thereafter, the visual acuity in both eyes was 20/20, and he was followed up in the retina clinic as an outpatient. Fluorescein angiography after treatment showed no macular edema or neovascularization (Figure 2).

Considering the association of CRM and arteriovenous malformations of the brain in approximately 24% of cases (compared with 0.2%-6% in the normal population)^(5)^, the patient also underwent contrast cranial magnetic resonance imaging, which showed no changes.

Archer et al.^(6)^ classified retinal arteriovenous communications into three groups. Group 1 includes smaller-caliber stable communications not respecting the midline, with normal angiographic flow and preserved capillary network. Group 2 includes larger-caliber communications, with a greater risk of thrombosis and break in the internal blood-retinal barrier. Group 3 includes cases with significant retinal disorganization and severe visual loss. Like most CRM cases, our case is compatible with Group 1 (Figure 2A).

However, even CRM cases similar to our case may present with a loss of visual acuity due to associated complications, such as occlusion of a central retinal artery branch^(6)^, bleeding, serous retinal detachment, or passage of a vessel through the foveola.

In conclusion, CRM is a rare diagnosis, seldom seen in general medical practice. However, these findings highlight the importance of identifying the disease; thus, ophthalmologists should be aware of CRM and monitor such patients regularly.
